# Quality of Life and Its Determinants in Older Adults with Intestinal Stomas: A Cross-Sectional Study

**DOI:** 10.3390/ijerph22030375

**Published:** 2025-03-04

**Authors:** Vitaliano de Oliveira Leite Junior, Giovanna Garcia Vieira, Keyliane Santos Lima, Silvana Mendes Costa, Ana Raquel Batista de Carvalho, Vanessa Moreira da Silva Soeiro, Ana Caroline Silva Caldas, Rafael Abreu Lima, Patricia Ribeiro Azevedo, Rosilda Silva Dias, Santana de Maria Alves de Sousa

**Affiliations:** Nursing Department, Federal University of Maranhão, São Luis 65080-805, Brazil; vitaliano.leite@discente.ufma.br (V.d.O.L.J.); giovannagarcia.sil@gmail.com (G.G.V.); keylianelima223@gmail.com (K.S.L.); silvana.mendes1412@gmail.com (S.M.C.); moreira.vanessa@ufma.br (V.M.d.S.S.); ana.caldas@ufma.br (A.C.S.C.); rafael.al@ufma.br (R.A.L.); patricia.azevedo@ufma.br (P.R.A.); rs.dias@ufma.br (R.S.D.); santana.sousa@ufma.br (S.d.M.A.d.S.)

**Keywords:** ostomy, quality of life, aged, chronic disease

## Abstract

Assessing the quality of life (QoL) of older adults with intestinal stomas is crucial to understanding the impact of body image and lifestyle changes in this often-underrepresented population. This study aims to evaluate the QoL of older adults with intestinal stomas and analyze the influence of sociodemographic and clinical factors on different QoL domains. This cross-sectional study analyzed the QoL of 47 older adults with intestinal stomas, using the City of Hope Quality of Life—Ostomy Questionnaire (COHQOL-OQ). Data were collected from the outpatient Orthotics and Prosthetics Program and analyzed using R software (version 4.3.1). The majority of participants were male (59.6%), with a mean age of 68.8 ± 5.6 years. Cancer was the leading cause of stoma creation (70.2%), with 83.0% having colostomies and 57.4% permanent stomas. The overall QoL score was 6.71 ± 1.64, with the lowest scores in the physical well-being domain (6.21 ± 2.31) and the highest in the spiritual well-being domain (7.91 ± 1.73). Significant associations were found between QoL and type of ostomy (*p* = 0.008), self-managed pouch changes (*p* = 0.050), and physical activity (*p* = 0.034). The study highlights the importance of autonomy and targeted interventions, including physical activity programs and psychoeducational support, to enhance QoL in this population. The findings provide valuable insights for healthcare professionals and policymakers, contributing to the development of evidence-based interventions for older adults with stomas.

## 1. Introduction

Healthcare systems face significant challenges, particularly regarding the increasing incidence of non-communicable diseases (NCDs) [1,2] such as colorectal cancer, which often necessitates the creation of intestinal stomas [3,4].

A stoma is defined as the surgical creation of an opening that establishes communication between an internal organ and the external environment for the purposes of respiration, nutrition, and the elimination of feces or urine [5]. Stomas can be temporary or permanent, depending on the diagnosis and severity of the underlying condition. Despite their importance for survival in severe clinical conditions, the creation of a stoma often leads to significant physical, emotional, and social challenges that require ongoing attention [6].

The prevalence of stomas is increasing as the older adult population grows, underscoring the relevance of this topic in the context of public health [7]. It is estimated that approximately 100,000 stomas are performed annually in the United States, with a significant proportion of these procedures occurring in individuals over 60 years of age [8]. In developing countries such as Brazil, estimates are less precise due to inconsistent records; however, it is believed that more than 207,000 people live with stomas [9,10]. Despite the growing number of older adults with stomas, there is a limited understanding of how different sociodemographic and clinical factors impact their quality of life, especially in low-resource settings [11].

Quality of life (QoL) is a multidimensional concept that reflects the perception of well-being across physical, psychological, social, spiritual, and cultural aspects [12]. For people with intestinal stomas, especially older adults, these dimensions are deeply affected. Physical well-being may be impaired by difficulties in stoma management, changes in diet, and skin integrity issues. Psychological well-being may be impacted by feelings of anxiety, depression, and shame. Social well-being may be compromised by limitations in interaction and returning to daily activities. On the other hand, the spiritual domain often emerges as a coping mechanism, playing a protective role in the resilience of these individuals [13,14].

International studies highlight that up to 50% of people with stomas may suffer from depression, and more than 40% report a negative impact on their social lives [11]. In Brazil, approximately 60% of older adults with stomas report difficulties in social adaptation and a significant decline in QoL after surgery [15]. These statistics emphasize the urgency of identifying key determinants that influence different QoL domains, as well as the specific needs of this population.

These issues can hinder social interaction and the emotional well-being of this population, highlighting the need for interventions that go beyond conventional medical treatment. In this context, adopting multidisciplinary support strategies is essential, integrating nursing care, psychological support, and social rehabilitation [13]. However, existing interventions often fail to consider the unique challenges posed by aging, such as multimorbidity, decreased functional capacity, and limited social support, which can further exacerbate the impact of an ostomy on daily life [11].

However, it is important to emphasize that although QoL is widely studied in people with stomas, most research focuses on adult populations [13,15,16,17,18], revealing a notable gap in the scientific literature concerning older adults with stomas [14]. Despite the high prevalence of this condition in aging populations, there is a lack of studies specifically analyzing the impact of sociodemographic and clinical variables on different QoL domains among older adults with stomas [16]. Given the vulnerability and unique challenges faced by this group, there is a need for research, especially in developing countries, that explores the specificities of this age group, aiming to develop interventions and policies better tailored to their needs [10].

The implementation of public policies aimed at improving the QoL of older adults with stomas is imperative. Investing in specialized and comprehensive care can promote advancements in well-being, autonomy, and independence for this population, making rehabilitation a viable goal for current healthcare systems.

Given the importance of this topic, this study aims to evaluate the quality of life of older adults with intestinal stomas in relation to sociodemographic and clinical factors.

## 2. Materials and Methods

This is a cross-sectional and analytical study with a quantitative approach, reported in accordance with the Strengthening the Reporting of Observational Studies in Epidemiology (STROBE) statement. The STROBE statement is a guideline aimed at ensuring methodological rigor and enhancing transparency in observational studies. It provides a comprehensive checklist to guide researchers in clearly reporting essential aspects of study design, data collection, analysis, and interpretation, thereby improving the reliability and reproducibility of the results [19].

A cross-sectional design was chosen as it enables the assessment of quality of life at a single point in time, making it appropriate for analyzing the influence of sociodemographic and clinical factors. While a longitudinal study could capture variations over time, the cross-sectional approach was deemed more viable considering participant accessibility and the structural limitations of the healthcare service. Additionally, this design is widely employed in research on quality of life in individuals with chronic health conditions, as it facilitates the examination of associations between different factors and quality of life outcomes [20,21].

Data collection took place within the Orthotics and Prosthetics Program, an outpatient service managed by the municipal public administration. The program operates under the Coordination of Healthcare for Persons with Disabilities of the Municipal Health Secretariat in São Luís, Brazil, providing multidisciplinary care for individuals with ostomies. The service is staffed by a specialized ostomy nurse, a psychologist, and a social worker, offering clinical follow-up, guidance on self-care, and support for social reintegration.

Patients enrolled in the program receive medical supplies such as ostomy bags and adjuvants, with distribution regulated based on individual needs. Access to these materials is centralized, with São Luís serving as a reference center for 174 municipalities, requiring many individuals to travel long distances to receive care and collect their supplies. The program also conducts regular follow-ups to monitor patients’ clinical conditions and update records, ensuring continuity of care.

The research was conducted from August 2019 to December 2022; however, data collection was suspended between March 2020 and July 2021 due to COVID-19-related restrictions, which limited participant recruitment and access to the outpatient service. The study population consisted of older adults registered in the Orthotics and Prosthetics Program, and data collection resulted in 47 participants, as the study adopted a census-based convenience sampling approach. The number of participants was influenced by service attendance patterns, hospitalization of potential participants, and challenges in reaching individuals who did not regularly access the service.

Older adults who met the following inclusion criteria were selected: receiving ostomy supplies from the outpatient service, being 60 years or older at the time of data collection, having had an intestinal stoma for at least six months, agreeing to participate by signing the Informed Consent Form (ICF), and being in good cognitive condition to participate in the study. This criterion was defined by the participant’s ability to understand the ICF and respond to the questionnaire coherently and independently. The research team clinically assessed this ability based on verbal interaction and the absence of signs of cognitive decline, such as disorientation or confusion [22]. Individuals who did not regularly attend the service to collect ostomy supplies and those who were hospitalized at the time of data collection were excluded.

Data collection was conducted using the registry of older adults enrolled in the Orthotics and Prosthetics Program who attended to collect the devices. They were invited to participate in the research, the ICF was read aloud, doubts were clarified, and after their agreement, they signed the form either in writing or by fingerprint. The research team responsible for administering the questionnaires consisted of two nurse professors, the research coordinators, three undergraduate students, and one postgraduate student, all trained over one month. The training included standardizing the approach, handling questions, and implementing strategies to minimize bias, ensuring the quality and consistency of responses.

The study applied three questionnaires: the first collected sociodemographic data, including sex, age, race, years of education, religion, origin, marital status, monthly income, type of residence, household characteristics, post-stoma employment, and support network; the second gathered clinical data such as causes of the stoma, type of stoma, duration, complications, bag changes, extra costs, difficulties, physical activity, smoking, alcohol consumption, and eating habits; and the third instrument used was the City of Hope Quality of Life—Ostomy Questionnaire (COHQOL-OQ), adapted and validated in Portuguese, which assesses the quality of life of people with stomas [23].

The COHQOL-OQ consists of 43 items divided into four subscales: physical well-being (items 1–11), psychological well-being (items 12–24), social well-being (items 25–36), and spiritual well-being (items 37–43). Each item was scored using a Likert scale ranging from 0 to 10, where zero represented the worst outcome and 10 the best. Certain items (13, 14, 16, 17, 20, 21, 31, 35, 36, and 38 to 43) had reverse scoring, meaning that higher values reflected poorer outcomes in those specific domains. The Likert scale is a psychometric response scale commonly used in questionnaires and is the most widely used scale in opinion research. For the reversed items in the questionnaire, point equivalence was performed statistically [23].

The COHQOL-OQ demonstrated strong internal consistency in this study, with Cronbach’s alpha values as follows: 0.92 for the overall instrument (very good reliability), 0.86 for the physical well-being domain (good reliability), 0.80 for psychological well-being (good reliability), 0.75 for social well-being (acceptable reliability), and 0.67 for spiritual well-being (moderate reliability). These values indicate that the instrument provides reliable assessments of quality of life in this population. Although the questionnaire is widely used in international research, its applicability across different age groups and cultural settings varies. However, previous studies have validated its use in Brazilian populations with ostomies, supporting its adoption in this study [23].

Statistical analysis was conducted using both parametric and non-parametric methods, depending on the nature of the variables. Categorical variables were analyzed using absolute frequencies and proportions, while numerical variables were described using means (±standard deviation—SD), minimum values, medians, maximum values, and interquartile ranges (IQR). The normality of numerical data was assessed using the Kolmogorov–Smirnov test, which indicated a non-normal distribution. Consequently, non-parametric tests were employed for comparisons between independent groups. Pearson’s chi-squared test was applied to categorical variables. A significance level of α = 0.05 was adopted, with results considered significant when *p* ≤ 0.05. Analyses were performed using Microsoft Excel^®^ 2023 and subsequently processed with R statistical software (version 4.3.1).

The project was reviewed and approved by the Research Ethics Committee of the Federal University of Maranhão under approval number 3.294.371/2019, in compliance with Resolution 466/2012 of the National Health Council (CNS), which establishes guidelines and regulatory standards for research involving human subjects in Brazil.

## 3. Results

The sociodemographic characteristics of the older people included in the study are described in Table 1. The majority of participants were male (59.6%) and aged between 60 and 69 years (57.4%), with a mean age of 68.8 years (SD ± 5.6, median: 68.0, IQR: 65.0–72.5). Mixed-race individuals were predominant (55.3%), while 31.9% had no formal education, and 38.3% had more than eight years of schooling. Most participants identified as Catholic (59.6%) and resided in the capital city (63.8%).

Regarding marital status, 53.2% were unmarried, although 68.8% reported having a support network. In terms of income, 87.4% received up to two minimum wages, and 85.2% did not engage in work activities after ostomy surgery.

Among the causes of ostomy, cancer was the predominant reason (70.2%), with most participants having a colostomy (83.0%) and a permanent stoma (57.4%). Regarding ostomy duration, 31.9% had lived with an ostomy for 21 years or more, and 55.3% reported no complications.

The majority of older people reported performing independent ostomy bag changes (68.1%), using adjuvants (63.8%), and incurring additional costs (61.7%). Most participants denied engaging in physical activity (80.9%), smoking (87.2%), and alcohol consumption (80.9%), as shown in Table 2.

In Table 3, the overall mean QoL score for older people with intestinal stomas was 6.71 (SD ± 1.64, median: 7.09, IQR: 5.52–7.96). Among the domains, spiritual well-being (SWB) presented the highest mean score (7.91 ± 1.73, median: 8.14, IQR: 7.14–9.14), followed by psychological well-being (PWB) (7.02 ± 1.81, median: 7.00, IQR: 5.92–8.58), social well-being (SoWB) (6.20 ± 1.94, median: 6.58, IQR: 4.46–7.71), and physical well-being (PhWB) (6.21 ± 1.94, median: 6.58, IQR: 4.46–7.71).

Regarding the reliability of the COHQOL-OQ questionnaire, the Cronbach’s alpha value for the overall instrument was 0.92, indicating very good reliability. The internal consistency values for each domain were 0.86 (PhWB), 0.80 (PWB), 0.75 (SoWB), and 0.67 (SWB).

The analysis of associations between sociodemographic and clinical variables and QoL domains, as measured by the COHQOL-OQ questionnaire, revealed statistically significant correlations, as presented in Table 4. Older women exhibited significantly higher scores in the PhWB domain compared to men (6.70 vs. 5.49; *p* = 0.018). Regarding ostomy etiology, older people with cancer had significantly higher scores in the SWB domain (*p* = 0.029).

Differences were also observed in relation to the type of ostomy, with older people with an ileostomy showing significantly lower QoL scores compared to those with a colostomy (*p* = 0.008), with significant differences in the PWB (*p* = 0.024), SoWB (*p* = 0.013), and SWB (*p* = 0.030) domains. Additionally, independent ostomy bag changes were associated with higher scores in the PWB (*p* = 0.028) and overall QoL (*p* = 0.050) domains.

The practice of physical activity was positively associated with higher scores in the PhWB (*p* = 0.018), SWB (*p* = 0.029), and overall QoL (*p* = 0.034) domains. Other variables analyzed, such as the presence of complications, stoma permanence, duration of the stoma, use of adjuvants, support network, and additional costs, did not present statistically significant associations with the evaluated QoL domains.

## 4. Discussion

The present study provided an understanding of the QoL of older people with intestinal stomas, highlighting sociodemographic and clinical factors that significantly influence the different dimensions of well-being in this population.

The sociodemographic profile was characterized by mixed-race men in their sixties, retired, with low income and education. Studies indicate that this group presents a risk profile, as men over the age of 60 tend to seek healthcare services less frequently and exhibit greater resistance to preventive care measures [24,25]. As a result, the progression of age is associated with increased health complications, particularly among those diagnosed with colorectal cancer, leading to greater dependency and higher healthcare demands [18].

The low income and education levels observed in this population are also risk factors for the development of NCDs. These conditions are directly linked to limited access to cancer prevention strategies, which represent the primary cause of stoma formation. Additionally, barriers to healthcare services and inadequate health literacy further compromise disease prevention, ultimately impacting QoL and increasing vulnerability to adverse health outcomes [24].

When analyzing the clinical characteristics of older people with stomas, the findings of this study align with previous research, which also report a predominance of colostomies associated with colorectal cancer diagnoses [18,26,27,28]. Data from the National Cancer Institute José Alencar Gomes da Silva reinforce this trend, demonstrating a progressive increase in mortality rates from intestinal cancers, particularly among older men [29]. In Maranhão, a state in northeastern Brazil, the estimated incidence is 6.88 cases per 100,000 men, making treatment more costly, complex, and challenging [30].

The duration of stoma use is another factor that may directly influence different QoL domains in older people with stomas. Individuals with long-term stomas tend to exhibit greater emotional and spiritual adaptation, which is reflected in the high scores for SWB (7.91) and PWB (7.02) in this study, as they gradually develop resilience and acceptance of their new life condition [31]. However, the lower scores in PhWB (6.21) and SoWB (6.20) indicate that difficulties related to mobility, pain, social interaction, and stigma persist, regardless of the adaptation period [32].

Older people with stomas reported a moderately positive perception of their QoL (overall mean score of 6.71), suggesting satisfactory adaptation to physical, emotional, and social demands, but with room for improvement when compared to other studies [16,18]. Scores close to 7 indicate that most participants are able to cope with the challenges posed by the ostomy, maintaining a good perception of well-being, especially in spiritual (7.91) and psychological (7.02) aspects. However, domains with lower scores, such as PhWB (6.21) and SoWB (6.20), highlight the ongoing difficulties in managing the physical and social aspects of living with an ostomy, which may negatively impact mobility, pain perception, and overall comfort [33].

Among the domains evaluated, SWB presented the highest scores, followed by PWB, SoWB, and PhWB, which had the lowest score. Studies conducted in Australia [34], Indonesia [35], and Brazil [36] emphasize the relevance of spirituality as a fundamental component for resilience and adaptation among ostomized patients. Spirituality provides meaning to the changes imposed by ostomy, encouraging self-care behaviors and psychological adaptation, making it a key protective factor in the coping process.

In contrast, PhWB is often compromised by functional limitations and concerns related to complications, which can be mitigated through targeted interventions, such as physical activity programs and specialized support [37]. SoWB, impacted by challenges in social interaction and reintegration, requires strengthening support networks and strategies to reduce stigma. Therefore, integrated interventions that address all QoL domains can promote comprehensive adaptation, significantly improving overall well-being [38].

The internal consistency of our study was considered acceptable, as assessed by Cronbach’s alpha. The Portuguese version of the COHQOL—Ostomy Questionnaire proved to be a valid and reliable tool for assessing the QoL of Brazilian older people with a stoma, indicating that the instrument is understandable and appropriate for this population [22].

The association between QoL domains and sociodemographic and clinical variables revealed significant relationships between PhWB, gender, and physical activity practice. These findings indicate that older women tend to maintain more effective self-care routines and possibly adapt more quickly to living with an ostomy, whereas older men frequently report greater difficulties, demonstrating a higher level of dependence in ostomy-related activities [33].

This discrepancy may reflect differences in perception of physical well-being between genders, suggesting that older women develop stronger coping strategies or resilience mechanisms regarding their physical condition [39]. Research suggests that women are more likely to seek social support and adhere to self-care practices, which can positively influence their perception of physical well-being [12,40]. These findings underscore the importance of gender-specific care approaches that address the distinct needs of men and women with stomas [40].

Furthermore, physical activity was associated with higher scores in PhWB, SWB, and overall QoL, reinforcing that engagement in physical exercise contributes to improved self-esteem, stress reduction, and the development of positive coping strategies—factors that benefit physical, mental, and spiritual health [41,42,43,44].

Women, in particular, exhibit greater adherence to self-care practices, including light and moderate physical exercises, which contribute to enhanced physical fitness, increased self-esteem, and the adoption of effective coping strategies. Conversely, older men more frequently report barriers to physical activity, such as fear of complications, which may negatively impact their perception of physical well-being [45,46].

These findings highlight the need for targeted interventions that consider gender differences, providing specialized support and promoting physical activity as a health strategy. Emphasis should be placed on personalized care programs that integrate health education and social support, fostering self-care practices, improving QoL, and facilitating adaptation among older people with stomas [47].

The predominance of men in this study may be attributed to the higher incidence of cancer—the primary cause of ostomy creation among participants. This is consistent with findings from previous research, which identify colorectal, sigmoid colon, and rectal neoplasms as the leading causes of colostomy formation [45].

The SWB domain emerged as an essential component in the adaptation of older people with intestinal stomas, showing high scores across different etiologies. Patients with cancer-related stomas exhibited significantly higher SWB scores, reinforcing that spirituality plays a crucial role, particularly in contexts of greater emotional and existential burden, such as cancer [46].

In settings where spirituality is deeply integrated into community practices and healthcare systems, it can serve as a robust coping mechanism, helping to mitigate the emotional, social, and QoL-related impacts of living with an ostomy. Therefore, it is essential to develop interventions that integrate continuous psychological support and coping strategies tailored to the individual needs of this population [14,15,47].

This study identified a significant association between colostomies and higher scores in the PWB, SoWB, SWB domains, and overall QoL compared to ileostomies. These differences may be attributed to the specific characteristics of each type of ostomy, as ileostomies are often associated with greater challenges in daily management, a higher frequency of leaks, and a more significant impact on body image [47,48].

Conversely, colostomies are perceived as less burdensome in terms of both physical and social demands, as they typically present a lower likelihood of leaks and odors, leading to greater confidence in social interactions and reduced stigma. These factors facilitate reintegration into daily activities and enhance social adaptation [32,49].

Additionally, these aspects have a positive impact on the psychological domain, as they help reduce anxiety and fear related to social exposure. The predictability of colostomy management supports self-care behaviors, enhancing the sense of autonomy and control over the condition, which contributes to improved self-esteem and more effective emotional adaptation [35,50].

The ability to independently change the ostomy bag was significantly associated with higher scores in PWB and overall QoL in this study. This highlights the importance of self-management in fostering a sense of control and confidence, which are often linked to lower anxiety levels and improved self-esteem [31,43].

Conversely, dependence on others for ostomy management can lead to feelings of insecurity, vulnerability, and anxiety, especially in situations that require autonomy, such as traveling or engaging in activities outside the home environment [51].

This lack of autonomy may result in embarrassment and intensify the perception of social stigma, limiting participation in social events and functional reintegration into society. Such factors are associated with significant negative impacts on the psychological and social domains of QoL, reinforcing the need for interventions that promote self-care and independence [35,47,52].

The literature underscores the importance of an interdisciplinary approach to ostomy care, emphasizing the role of educational interventions led by ostomy care nurses, social workers, and psychologists. Ostomy care nurses play a central role in empowering patients for independent ostomy management, contributing not only to improving technical knowledge but also to enhancing physical, psychological, and social aspects of QoL [33,53].

In addition to this role, social workers help identify and overcome structural barriers, such as difficulties in accessing ostomy materials and healthcare services, while also connecting patients to community resources that promote support and inclusion.

Psychologists, in turn, play a critical role in strengthening emotional well-being, by assisting with condition acceptance, developing coping strategies, and reducing anxiety, thus enhancing resilience and adaptation [5,37,50].

In the context of developing countries such as Brazil, the challenges faced by older people with intestinal stomas are even more pronounced due to structural limitations in healthcare systems, restricted access to ostomy supplies, and a shortage of specialized professional support [36].

Studies conducted in low-resource settings consistently highlight the scarcity of infrastructure and resources as significant barriers to effective ostomy care and the maintenance of QoL [15,24,40]. Additionally, socioeconomic factors such as low income and limited education may further hinder access to essential information and healthcare services, increasing the risk of complications and negatively impacting overall well-being [37].

## 5. Limitations

The main limitation of this study was the interruption of data collection due to the COVID-19 pandemic, which required adaptations in recruitment and impacted sample representativeness. The study adopted a census-based convenience sampling approach, limited to participants who remained engaged with the service during and after the pandemic. This may have restricted the generalizability of the findings, as individuals with reduced healthcare access or additional challenges during this period may not be adequately represented [54].

Despite this limitation, the study provides a relevant depiction of the challenges faced by older people with stomas during the pandemic. The findings reflect barriers to healthcare access, changes in social interactions, and psychological adjustments, contributing to the understanding of ostomy management under exceptional circumstances.

Extending data collection beyond 2022 was avoided to preserve sample comparability, as mass vaccination, healthcare system restructuring, and post-pandemic adaptations could have significantly modified the characteristics of the study population. Resuming recruitment under different conditions would have compromised data consistency and introduced biases, affecting the validity of the results.

Despite these constraints, the study was methodologically rigorous, employing validated assessment tools and robust statistical analyses, ensuring the reliability of the findings.

## 6. Conclusions

This study highlights that older people with intestinal stomas experience a moderately positive quality of life, with the SWB domain being the most preserved, while PhWB remains the most compromised. Key factors associated with better QoL scores include ostomy type, independent self-care in bag changes, and engagement in physical activity, reinforcing the importance of autonomy, multidisciplinary support, and structured care strategies.

These findings emphasize the need for healthcare policies and interventions that support self-care, psychosocial assistance, and social reintegration programs. Given the aging population and increasing incidence of ostomies, expanding access to comprehensive, multidisciplinary care is essential to optimize well-being and independence in this group.

Future longitudinal studies are necessary to assess long-term adaptations, particularly considering sociocultural differences and variations in healthcare systems. Understanding how different social and health contexts shape the experience of living with an ostomy will allow for tailored interventions and policies that better address the needs of diverse populations.

## Figures and Tables

**Table 1:** Sociodemographic characteristics of older people with intestinal stomas registered in the Orthotics and Prosthetics Service in São Luís–MA, 2023 (*n* = 47).

Sociodemographic Variables	*n*	%
Gender		
Male	28	59.6
Female	19	40.4
Age group ($\bar{x}$ = 68.8 ± 5.6)		
60–69 years	27	57.4
70–79 years	19	40.4
≥80 years	1	2.1
Skin color/Race		
White	17	36.2
Brown	26	55.3
Black	4	8.5
Education level		
0 years	15	31.9
1–4 years	5	10.6
5–8 years	9	19.1
>8 years	18	38.3
Religion		
Catholic	28	59.6
Evangelical	16	34.0
Other	1	2.1
None	2	4.3
Place of residence		
São Luís	30	63.8
Other municipality	17	36.2
Marital status		
With spouse	22	46.8
Without spouse	25	53.2
* Monthly income (MW)		
<1 minimum wage	21	44.7
1–2 minimum wages	20	42.6
2–4 minimum wages	3	6.4
>4 minimum wages	3	6.4
Post-stoma employment		
Yes	7	14.9
No	40	85.1
Support network		
Yes	32	68.1
No	15	31.9

* minimum wage reference in 2022: RD 1212.00/USD 497 in purchasing power parity (https://ilostat.ilo.org/topics/wages/ accessed on 15 January 2025).

**Table 2:** Clinical data of older people with intestinal stomas registered in the Orthotics and Prosthetics Service in São Luís–MA, 2023 (*n* = 47).

Clinical Variables	*n*	%
Cause of stoma		
Cancer	33	70.2
Non-oncological intestinal obstruction	5	10.6
PFA/PMW *	2	4.3
Other	7	14.9
Type of ostomy		
Colostomy	39	83.0
Ileostomy	8	17.0
Ostomy permanence		
Temporary	20	42.6
Permanent	27	57.4
Duration of ostomy		
Less than 1 year	4	8.5
1 year	6	12.8
2 to 5 years	10	21.3
6 to 10 years	7	14.9
11 to 20 years	5	10.6
21 years or more	15	31.9
Change of ostomy bag		
Self change	32	68.1
Other person	15	31.9
Complication		
Yes	21	44.7
No	26	55.3
Use of adjuvants		
Yes	30	63.8
No	17	36.2
Additional cost		
Yes	29	61.7
No	18	38.3
Physical activity		
Yes	9	19.1
No	38	80.9
Smoking		
Yes	6	12.8
No	41	87.2
Alcohol consumption		
Yes	9	19.1
No	38	80.9

* PFA—perforation by firearm/PMW—perforation by melee weapon.

**Table 3:** Mean values of domains and Cronbach’s alpha of COHQOL-OQ among older people with intestinal stomas in São Luís–MA, 2023 (*n* = 47).

COHQOL-OQ Domains	Mean	SD *	Minimum	Maximum	Cronbach’s α
Physical (PhWB)	6.21	±2.31	0.90	9.36	0.86
Psychological (PWB)	7.02	±1.81	1.92	9.69	0.80
Social (SoWB)	6.20	±1.94	2.50	9.17	0.75
Spiritual (SWB)	7.91	±1.73	1.43	10.00	0.67
Total Quality of Life (QoL)	6.71	±1.64	2.63	9.13	0.92

* standard deviation.

**Table 4:** Association between sociodemographic and clinical data and COHQOL-OQ scores of older people with intestinal stomas in São Luís–MA, 2023 (*n* = 47).

Variables	Physical (PhWB)		Psychological (PWB)		Social (SoWB)		Spiritual (SWB)		Total QoL Mean	
	Mean	*p*-Value	Mean	*p*-Value	Mean	*p*-Value	Mean	*p*-Value	Mean	*p*-Value
Gender										
Female	5.49	0.018 *^u^*	6.85	0.468	6.48	0.441	8.38	0.128	6.64	0.555
Male	6.70		7.13		6.00		7.58		6.75	
Ostomy etiology										
Cancer	6.46	0.069	7.24	0.567	6.28	0.705	8.03	0.029 ^x^	6.88	0.413
Obstruction	5.75		6.45		4.83		6.29		5.75	
PFA/PMW	6.32		8.08		7.54		7.86		7.43	
Other	5.35		6.10		6.37		8.47		6.38	
Type of ostomy										
Colostomy	6.56	0.072	7.35	0.024 *^u^*	6.50	0.013 *^u^*	8.20	0.030 *^u^*	7.03	0.008 *^u^*
Ileostomy	4.55		5.39		4.69		6.46		5.14	
Ostomy bag change										
Self change	6.55	0.110	7.41	0.028 *^u^*	6.53	0.060	8.12	0.298	7.04	0.050
Other person	5.50		6.18		5.47		7.45		6.00	
Ostomy Permanence										
Temporary	6.01	0.897	6.83	0.401	5.72	0.143	7.51	0.072	6.40	0.261
Permanent	6.36		7.16		6.55		8.20		6.94	
Duration of ostomy										
Less than 1 year	6.98	0.538	7.60	0.256	6.19	0.096	7.04	0.345	7.04	0.396
1 year	6.48		6.71		5.68		8.05		8.05	
2 to 5 years	6.48		7.31		6.61		8.60		8.60	
6 to 10 years	5.81		7.02		6.69		7.80		7.80	
11 to 20 years	7.33		7.31		6.98		8.49		8.49	
21 years or more	5.55		6.70		5.63		7.48		7.48	
Use of Adjuvants										
Yes	5.92	0.363	6.78	0.204	5.99	0.387	7.87	0.946	6.50	0.391
No	6.78		7.47		6.58		7.97		7.11	
Support Network										
Yes	6.22	0.882	7.11	0.545	6.51	0.123	8.07	0.160	6.85	0.243
No	6.21		6.83		5.53		7.55		6.40	
Physical activity										
Yes	7.58	0.018 *^u^*	7.63	0.239	7.05	0.105	8.83	0.029 *^u^*	7.63	0.034 *^u^*
No	5.89		6.87		5.99		7.69		6.49	

^x^ *p*-value calculated by Pearson’s chi-squared test. *^u^ p*-value calculated by Mann–Whitney test.

## Data Availability

The original contributions presented in this study are included in the article. Further inquiries can be directed to the corresponding author.
